# Effects of varying tillage practices and weed control methods on the efficacy of infiltration models

**DOI:** 10.1371/journal.pone.0293507

**Published:** 2024-01-25

**Authors:** Thomas Atta-Darkwa, Austin Asare, Killian Asosega Asampana, Maxwell Budu, Alex Agbeshie Amerh, Samuel Asomaning Kwesi, Enoch Bessah, Prosper Achaw Owusu, Andrew Quansah, Emmanuel Nyantakyi Kwasi, Ebenezer K. Siabi

**Affiliations:** 1 Department of Agricultural and Bioresources Engineering, University of Energy and Natural Resources, Sunyani, Ghana; 2 Department of Environmental Management, University of Energy and Natural Resources, Sunyani, Ghana; 3 Department of Mathematics and Statistics, University of Energy and Natural Resources, Sunyani, Ghana; 4 Department of Agricultural Engineering, Ho Technical University, Ho, Ghana; 5 Department of Horticulture and Crop Production, University of Energy and Natural Resources, Sunyani, Ghana; 6 Department of Agricultural and Biosystems Engineering, Kwame Nkrumah University of Science and Technology, Kumasi, Ghana; 7 Department of Computer and Electrical Engineering, University of Energy and Natural Resources, Sunyani, Ghana; 8 Department of Civil and Environmental Engineering, University of Energy and Natural Resources, Sunyani, Ghana; 9 Earth Observation Research and Innovation Center (EORIC), University of Energy and Natural Resources, Sunyani, Ghana; ICAR Research Complex for Eastern Region, INDIA

## Abstract

Agricultural land preparation and weed control techniques are essential farm management tools that affect the dynamics of soil water infiltration and the estimation accuracy of infiltration models. To analyse the interaction effect of tillage and weed control methods on the changes in soil physical properties and the efficacy of infiltration models, an experiment was conducted on a sandy clay loam forest ochrosol at Hodzo near Ho in Ghana. Four tillage systems (No Tillage [NT], Reduced Tillage [RT], Plough + Harrow + Ridging [PHR], and Deep Tillage + Plough + Harrow + Ridging [DPHR]) and three weed control methods (Hoeing [H], Machete [MAT] and No Weeding [NW]) were employed. The study also tested the reliability of the models (Kostiakov, Philip, and Horton) using the goodness of fit statistical criteria: Root mean squared error (RMSE), Mean absolute error (MAE), Coefficient of determination (R^2^), and Nash-Sutcliffe efficiency (NSE). The results show that conservation tillage systems (CsT) and conventional tillage systems (CT) with MAT weeding treatments recorded the highest moisture content across the studied soil profile, especially for NT x MAT (11.189%) which was significant (p < 0.05) in the 15–30 cm layer; the lowest were observed in the CsT and CT with H weeding interactions, especially for the DPHR x H (8.086%). Comparing the interaction effect on the soil infiltration, the highest mean infiltration rate was significant (p < 0.05) under the NT X H treatment combination whilst the lowest infiltration rate was recorded in the DPHR X H and PHR X NW treatment combinations. The efficiency of the fitting models (Kostiakov *>* Horton *>* Philip) highly prioritised the soil tillage operations and weed management under the treatments DPHR x MAT > DPHR x NW > DPHR x H > RT x MAT > PHR x NW > PHR x MAT > NT x NW > RT x MAT > PHR x H > RT x H > NT x MAT > RT x NW > NT x H in that order. The trend shows that the increase in tillage intensity and the decrease in weed management intensity induce the quality of the estimation process and vice versa. The study, therefore, identified the use of machete (MAT) with DPHR under the Kostiakov model as the efficient land management for modelling the cumulative infiltration characteristics of the sandy clay loam ochrosols of the study area.

## 1.0 Introduction

Tillage may be classified as conventional, reduced (minimum), or conservation if it preserves crop mulch covering >30% of the soil surface after planting. Soil tillage, as an important land management technique in agricultural production, can affect the soil’s physical qualities that are crucial for crop growth [1, 2]. Among the crop production factors, tillage can make up to 20% of the elements that affect crop productivity [3]. Improvements in soil moisture storage, root penetration and water infiltration, weed control, and delivery of nutrients through the quick breakdown of organic matter are considered the greatest, valuable contributions of tillage to crop production [4, 5]. However, long-term conventional tillage (CT) practices may affect the soil structure, aggregate stability, moisture storage, porosity, soil micro- and macro-fauna, soil erosion, organic matter, and environmental quality by fast-tracking production costs and greenhouse gas emissions [6, 7], in addition to altering soil qualities by affecting percolation and soil infiltration rates. Other studies have reported several advantages of conservation tillage (CsT) involving minimal soil tillage for crop production–advantages such as reduction in soil erosion and surface runoff [8, 9]; enhancing soil structure and moisture storage [10]; improvement of aggregate stability and retention of soil organic matter [11–13]; and improving water infiltration and minimization of soil temperature fluctuations [14, 15]. Tillage affects the soil water’s hydraulic and osmotic potentials, while it also increases surface water retained in depressions and soil infiltrability [16, 17]. Soil water infiltration is affected by several factors such as tillage and residue retention, roughness of surface cover, soil density and porosity, organic carbon content, aggregates stability and size, and soil moisture content [18]. Soil water infiltration processes play a crucial role in the analysis of irrigation and drainage systems design, diffuse groundwater recharge evaluation, overland flow, soil erosion evaluation, subsurface flow and investigation of pollution transport, and hydrologic systems design and modelling [19]. The infiltration characteristics of a field that is being used for agricultural production are essential to the effective use of irrigation water by improving water distribution efficiency. Given that agriculture consumes the most water globally, especially in sub-Saharan Africa and other developing nations, even a slight improvement in efficient use of water can result in significant freshwater savings [20, 21]. The growing significance of infiltration processes in managing the available limited freshwater resources resulted in the derivation of infiltration models and equations for simulating and predicting infiltration rate and cumulative infiltration amount [20]. For extremely accurate modelling of soil infiltration behaviour, a strong data-driven infiltration model is required [22]. The infiltration models used in the estimation of infiltration rate and cumulative infiltration amount have been categorised into empirical, semi-empirical, and physically based or deterministic models [23]. The empirical models include the Kostiakov model [24], the Mezencev model [25], the Revised Modified Kostiakov [26], the Novel model [27] and the Collis-George model [28], which are derived using laboratory or field data. The semi-empirical models are established from the infiltration rate-cumulative infiltration relationship and the continuity equation also includes the Horton model [29], Holtan [30], Overton [31], and Singh and Yu [32]. The deterministic models which are based on the Darcy’s law and basic laws of conservation of mass also include the Green and Ampt model [33], the Philip model [34], the Brutsaert model [35], the Smith and Parlange model [36] and the Swartzendruber model [37]. Recent researches have examined the effects of land use/land cover changes and land management practices on the features of soil infiltration [9, 16, 18, 20, 38–40].

Shukla et al. [38] found out how tillage operations affected the infiltration rate and cumulative infiltration amount of the silty clay loam soil. According to their research, the no-tillage operation had the highest steady infiltration rate and cumulative infiltration amount (27.26 cm/h, 90.6 cm), compared to the chisel plough and mouldboard plough tillage practices. Shao and Baumgartl [41] employed a rainfall simulator to produce infiltration data on some experimental fields of eastern Australia and observed that the Holtan and Horton models outperformed the Philip and Green Ampt models in predicting infiltration rates under various soil conditions under various vegetation covers.

Carvalho et al. [9] evaluated the effects of tillage systems and growth stages of corn on soil runoff and infiltration rates. They reported that the Horton model was the most suitable in simulating soil water infiltration rate under the assessed conditions. Osanyinpeju and Dada [42] also found that the infiltration capacity of their studied soil increased from no-tillage (24.40 cm/h), followed by disc plough tillage (32.30 cm/h) and disc harrow tillage (39.40 cm/h), in that order.

The Kostiakov, Philip, and Horton models were used by Atta-Darkwa et al. [22] to calculate the infiltration rate for conventional and conservation tillage operations. They discovered that the studied sandy clay loam soil’s infiltration capacity increased in the following orders: DPHR, PHR, RT, and NT; with the Kostiakov model more accurately predicting the observed cumulative infiltration depth than the Philip and Horton models under the various tillage operations.

Knowledge of soil water infiltration before any agricultural land preparation operation is essential for effective agricultural activities [43]. The direct and indirect effects of weed management, which can be both beneficial and bad, have an effect on soil health and crop productivity [44]. Soil surface disturbance differs under different tillage, hand weeding, and chemical control methods [45]. Much research have been conducted to assess the influence of tillage on soil physical properties and the effectiveness of infiltration models. However, to the best of our knowledge, the interactive effect of different tillage operations and mechanical weed control methods (options—Hoeing, Machete, and No weeding) on modelling soil water infiltration has not been investigated.

Therefore, the objective of this study was to analyse how different tillage techniques and weed control methods affect the characteristics of sandy clay loam soil’s infiltration, as well as how well the models developed by Kostiakov, Philip, and Horton predict cumulative infiltration amount.

## 2.0 Materials and methods

### 2.1 Study area

**Fig 1:**
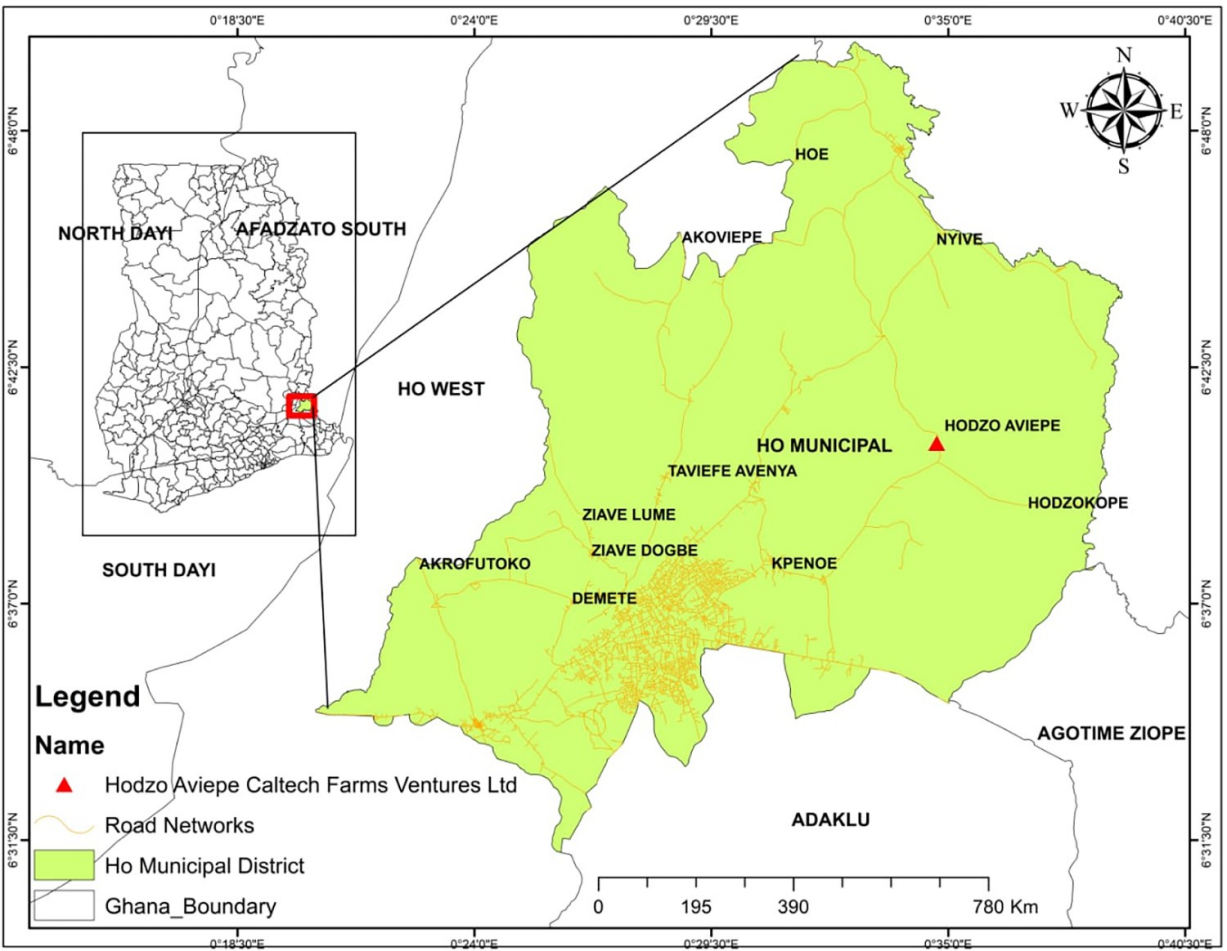
Map of Volta regional location showing study site [Source: Authors own construct].

The test was carried out at CALTECH Farms in Hodzo, near Ho. The area has an elevation between 60 and 152 meters above sea level and is located between latitudes 6° 207 N and 6° 55 N and longitudes 0° 127 E and 0° 53 E [46]. The dry Harmattan winds from the Sahara and the southwest monsoons from the South Atlantic both have a significant impact on the study area’s tropical climate (Fig 1). The yearly average temperature ranges from 16.5°C to 37.8°C, with the monthly mean temperature falling between 22°C and 32°C. The region experiences two seasons of rainfall each year: the major season which runs from mid-March to June, and the minor season which starts in July and runs through to November. The yearly average rainfall figures range from 1,168 mm to 2,103 mm, respectively. The month of November has the lowest average monthly rainfall with 20.1 mm, while June has the most rainfall per month with a mean value of 192 mm. The relative humidity is often about 80%. The majority of the hills in the region are covered by a moist semi-deciduous forest, while the remainder of the Municipality is covered by savannah woodland and is drained by the Volta Lake. The two types of soils are classified as savannah soils and the forest ochrosols, lethosols, and intergrades found in the wetter northern and mountainous areas. Prior to the investigation, the site had not been cropped for roughly eight (8) years. Cassava and maize were intercropped there prior to the fallow period. Tables 1 and 2 illustrate, respectively, selected physico-chemical soil parameters and weather data for the experimental site.

**Table 1:** Selected Soil physico-chemical properties at the experimental site.

	Soil Depth		
Soil Property	0–15 cm	15–30 cm	30–60 cm
Sand (%)	67.13	76	75.72
Silt (%)	10.37	1.5	1.77
Clay (%)	22.5	22.25	22.25
Organic Carbon (%)	0.93	0.19	1.29
EC (μs/cm^3^)	279	397	87.4
pH H_2_O	6.59	6.31	6.22
Total N (%)	0.13	0.07	0.05
Ca (cmol kg^-1^)	8.73	5.12	3.63
Mg (cmol kg^-1^)	1.77	0.99	0.65
K (cmol kg^-1^)	0.52	0.14	0.15
Available P (mg kg^-1^)	14.44	6.43	7.02
Na (cmol kg^-1^)	0.2	0.18	0.2

**Table 2:** Weather data for Ho (Temperature, relative humidity, rainfall and evapotranspiration-July 2017 to June 2018).

Month	T_max_ (°C)	T_min_ (°C)	T_mean_ (°C)	Rel. Humidity (%)	Rainfall (mm)	ET0 (mm/m)
July	29.4	22.7	26.05	85	145.8	66
August	28.7	22.4	25.55	88	56.7	66.2
September	30.3	22.6	26.45	86	94.1	78.7
October	32.4	22.4	27.4	81	81.3	88.3
November	33.1	23.1	28.1	76	41.2	109
December	33.6	23.2	28.4	72	31	156
January	34.6	22.5	28.55	69	0	204.4
February	35.6	24.8	30.2	77	19.4	159.6
March	34.8	24.1	29.45	76	59.9	151
April	34.3	24.5	29.4	75	44.9	138.5
May	32.4	23.7	28.1	78	150.7	103.7
June	30.9	23	26.95	81	119.3	75.8

T_max_ (°C)- Maximum Temperature; T_min_ (°C) -Minimum Temperature; Tmean−Mean Temperature; ET_0_ -Evapotranspiration

### 2.2 Experimental design and treatments

A split-plot with three replications was used as the experimental design, which was set up in a randomized complete block design. Tillage was the primary factor on the major plots, and weed control was the primary factor on the sub-plots. The sub-plots measured 8 m by 5 m with 1.2 m buffers, whereas the major plots were 15 m by 8 m with 2 m buffers between them. Twelve (12) sub-plots and 4 main plots were present. The systemic herbicide, Glyphosate 360 SL, was sprayed over the entire field at a rate of 10 ml per litre of water. After two weeks, conventionally tilled plots were hacked clean using machetes and then stumped with mattocks before being ploughed. For those that utilised conservation tillage, there was no stumping. In order to mimic a roller crimper, a wooden pole was used. The following were the tillage treatments: Ploughing + Harrowing + Ridging (PHR), No Tillage (NT), Reduced Tillage through heaping (RT), and Deep Tillage + Ploughing + Harrowing + Ridging (DPHR). One (1) pass per tillage treatment was used. The three weed management methods were hand hoeing, weeding with a machete, and no weeding. Weeding was done 2 Months after Planting (MAP), 4 MAP, 6 MAP, and 8 MAP. One (1) pass per tillage treatment was used. The tillage treatments’ typical depths are as follows: 60 cm for deep tillage, 55 cm for a disc plough, 55 cm for harrowing, and 55 cm for ridging.

The undisturbed soil samples collected at depths of 0–15 cm, 15–30 cm, and 30–60 cm were chosen to reflect the cutting depth of the tillage implements.

### 2.3 Management of field crops

The stems of Bosome Nsia or Dzinu Ade cassava variety were acquired from a farmer in the Ho Municipality and unhealthy ones screened out. Cuttings from healthy sticks, 20 to 25 cm long were planted with about a third of the cuttings above ground at angle of 45° using a machete (Hauser et al., 2014). A 1 m by 1 m plant spacing was adopted (120 stakes on each main plot), giving 10,000 plants per hectare. There was no fertilizer application, mechanical or chemical disease and/or pest control in this experiment, which was also entirely rain-fed. After cutting off the tops, the tubers were harvested by hand lifting.

### 2.4 Measurement of field infiltration

The infiltration capacity was measured using a double ring infiltrometer, which contains inner and outer rings with diameters of 30 cm and 60 cm, respectively. After carefully removing any small obstructions at the surface that would obstruct smooth insertion, the inner ring was inserted first, then the outer ring, 5 cm into the earth. Steel tape and a spirit level were used to check for concentricity and to make sure both rings were at the same depth. To establish an equilibrium hydraulic head within and outside of the smaller cylinder, both cylinders are filled with water to the same height. A ruler was put into the inner cylinder at a height of 10 cm to measure the drop in water level over a certain period. To maintain approximately equal hydraulic heads throughout the observation time, both rings were filled as the water levels dropped.

The process was repeated several times at 1, 2, 5, 10, 15, 20, 25, 30 minute intervals where constant or almost constant values were documented as the basic infiltration rate. The parameters of the infiltration models were estimated using infiltration data collected in the field.

### 2.5 Determination of dry bulk density and moisture content

Each plot had three sets of undisturbed soil samples collected at depths of 0–15 cm, 15–30 cm, and 30–60 cm. To determine the dry bulk density and moisture content, the soil in the core sampler was weighed and then dried in an oven at 105°C for 24 hours [47]. Dry bulk density (*ρ*_*d*_) was determined and displayed in Mgm^-3^ as follows:

$$\rho_{d}=\frac{M_{3}-M_{1}}{V_{t}}$$ (1)

Soil moisture content (*θ*_*m*_) was also calculated on mass bases and displayed as a percentage of moisture.

$$\theta_{m}(\%)=\frac{M_{2}-M_{3}}{M_{3}-M_{1}}\times 100$$ (2)

where:

M_1_ = Mass of container

M_2_ = Mass of the container and moist soil

M_3_ = Mass of the container and oven-dried soil

*V*_*t*_ = Volume of the core sampler

### 2.6 Model parameters and infiltration rate estimation

The field measured infiltration values in this investigation were modified to fit the models developed by Kostiakov, Philip and Horton. The infiltration models selected for this study were chosen owing to their usefulness and popularity in previous studies [9, 21].

### 2.6.1 Kostiakov’s model

The infiltration rate equation was presented by Kostiakov as an empirical equation, and it is written as

$$i_{p}=abt^{b-1}$$ (3)

To determine the cumulative infiltration amount *i*_*p*_, Eq (3) is integrated.

$$I_{p={at}^{b}}$$ (4)

where *i*_*p*_ *and I*_*p*_ are infiltration rate (mm/h) and cumulative infiltration (mm), respectively, and t = time since the measurement of infiltration began (h). Constants a and b are affected by the initial soil properties, where a > 0 and 0 < b < 1.

ln (*I*_*P*_) is plotted against *ln*(*t*) to estimate the Kostiakov model’s parameters. The slope and intercept of the best-fit straight line determined from the plot are given as *b* and *lna*, respectively.

The Kostiakov model is widely favored for surface irrigation applications due to its straightforwardness, ability to accommodate various infiltration data, and its flexibility regarding water application methods, in contrast to certain other models. According to this model, it assumes that at the outset, the infiltration rate is extremely high and gradually diminishes to near-zero levels over an extended period [32]. However, it’s worth noting that the Kostiakov model excels in describing the initial phases of the infiltration process but tends to lose accuracy as time progresses, as noted by Philip [34].

#### 2.6.2 Horton’s model

Cumulative infiltration over time is expressed as an exponential decay by the following three-parameter equation of Horton:

$$I_{p}=i_{c}t+\frac{\left(i_{0}-i_{c})({1-e}^{-K_{h}t}\right)}{K_{h}}$$ (5)

To determine the infiltration rate, Eq (5) is differentiated as follows:

$$i_{p=}i_{c}+{(i}_{0-}i_{c})e^{K_{h}t}$$ (6)

where *i*_*p*_ = initial infiltration capacity (mm/h) at *t* = 0; *i*_*c*_ = steady state infiltration rate (mm/h); and *K*_*h*_ = Horton’s decay coefficient is affected by soil properties and vegetative cover. *ln*(*i*_*p*_−*i*_*c*_) is plotted against *t* to determine Horton’s model’s parameters. The slope and intercept of the best-fit straight line determined by the plot are given as *ln*(*i*_0_−*i*_*c*_) and *K*_*h*_, respectively.

The Horton equation remains applicable solely when the rate of rainfall consistently surpasses the soil’s infiltration capacity throughout the entire rainfall-infiltration process. An additional limitation is the difficulty in incorporating changes in antecedent conditions [30]. However, it exhibits a generally favorable alignment with in situ data and effectively captures the fundamental characteristics of soil, as evidenced by the fact that three of its parameters are derived from experimental measurements.

#### 2.6.3 Philip’s model

To answer the one-dimensional Richards equation, Philip suggested a grounded two-term physically based infiltration model. The following is the mathematical formulation of Philip’s equation:

ip=12st−12+K (7)

Eq (7) was integrated to produce the equation for cumulative infiltration:

Ip=S2t12+Kt (8)

where *i*_*p*_ = Infiltration capacity (mm/h), S = soil water sorptivity which depends on initial soil moisture content and water diffusivity, and, as enumerated by the law Darcy, K = Hydraulic conductivity at saturation. Plotting *i*_*P*_ against $t^{-\frac{1}{2}}$ yields the Philip model’s parameters. The best-fit straight line obtained from the plot gives $\frac{s}{2}$ and *K* as the slope and intercept, respectively.

Among the physically-based infiltration models, the Philip’s model stands out as particularly appealing due to its computational simplicity, allowing time to be explicitly expressed as a function of ’cumulative infiltration’ or vice versa [37]. However, there’s an inherent truncation error associated with parameter K in this model, arising from the use of only the first two terms in the infinite series solution.

### 2.7 Statistical analysis

#### 2.7.1 The efficiency of the models’ predictions

The efficiency of the models’ predictions was evaluated using Coefficient of Determination (R^2^), Root Mean Squared Error (RMSE), Nash-Sutcliffe Efficiency (NSE) and Mean Absolute Error (MAE) statistical indices.

*2*.*7*.*1*.*1 Coefficient of determination (R*^*2*^*)*. The coefficient of determination determines how much of the observed distribution is accounted for by the estimated value’s distribution. The formula used to compute the coefficient of determination is:

$$R^{2}=\frac{n\sum x_{i}^{\prime }x_{i}-(\sum x_{i}^{\prime })(\sum x_{i})}{\sqrt{n(\sum {\left(x_{i}^{\prime }\right)}^{2})}-{\left(\sum x_{i}^{\prime }\right)}^{2}\sqrt{n(\sum x_{i}^{2})}-{\left(\sum x\right)}^{2}}$$ (9)

*2*.*7*.*1*.*2 Root mean square error (RMSE)*. The standard deviation of the variations between the measured and anticipated values is represented by the root mean square error values. The RMSE is calculated by

$$RMSE=\sqrt{\frac{1}{N}(\sum_{i=1}^{n}{\left(x_{i}^{\prime }-x_{i}\right)}^{2})}$$ (10)

where $x_{i}^{\prime }$ is the simulated and *x*_*i*_ is the observed data of the cumulative infiltration amount, and *N* denotes the number of observations.

*2*.*7*.*1*.*3 Mean absolute error (MAE)*. The mean absolute error is the difference in absolute terms between the measured value and the simulated value. The absolute error is estimated as

$$MAE=\frac{1}{N}\sum_{i=1}^{n}\left|x_{i}^{\prime }-x_{i}\right|$$ (11)

where $x_{i}^{\prime }$ is the estimated and *x*_*i*_ is the measured value of the cumulative infiltration amount, and *N* is the number of observations.

*2*.*7*.*1*.*4 Nash-sutcliffe efficiency (NSE)*. This is the average difference between the measured values of the cumulative infiltration and the estimated values. The NSE is evaluated by

$$NSE=\frac{\sum_{i=1}^{n}{\left(x_{i}-x_{i}^{\prime }\right)}^{2}}{\sum_{i=1}^{n}{\left(x_{i}-\bar{x}\right)}^{2}}$$ (12)

where $x_{i}^{\prime }$ is the estimated and *x*_*i*_ are the measured values of the cumulative infiltration amount, and *N* denotes the number of observations.

### 2.8 Data analysis

Utilizing both descriptive and inferential statistics, the research data was examined. R-Statistical software, version 3.6.0, was used to analyse the data [48]. Tables and graphs were created using descriptive statistics to characterise the physical characteristics of the soil in terms of tillage and weed control, such as the dry bulk density, moisture content, and infiltration rate. ANOVA and general linear models were employed with inferential statistics to ascertain the treatment and interaction effects of weed management and tillage on soil physical parameters. One-way analysis of variance (ANOVA) was used to evaluate the average infiltration rate among the various tillage and weed control schemes in order to see if there were any significant differences at the 5% level of significance. The Fisher Multiple comparison post hoc test was used to distinguish between the means.

Furthermore, using the Fisher Multiple comparison post hoc test, the predicted cumulative infiltration amount of the soil was compared to the actual cumulative infiltration amount under the Kostiakov, Philip and Horton infiltration models.

## 3.0 Results and discussion

### 3.1 Effect of tillage and weed control methods’ interaction on dry bulk density

Table 3 summarizes the findings on the interaction between weed management techniques and tillage on soil dry bulk density. To a greater extent, dry bulk density increased with depth across all treatment combinations. Throughout the experiment, CsT and CT systems with MAT weeding method had lower bulk densities and statistically significant interaction effects (p < 0.05) at 0–15 cm and 30–60 cm depths, compared to CsT and CT with H and NW combinations which gave slightly higher bulk densities but did not have a significant interaction effect (p > 0.05) across all depths. Comparing the significant interaction effect of the tillage treatments with MAT, the soil bulk density of RT x MAT, PHR x MAT, DPHR x MAT were higher relative to NT x MAT at 2.72%, 1.49% and 7.9% for the 0–15 cm layer and 2.97%, 1.76% and 3.45% for the 30–60 cm layer, respectively. Among the tillage and weed control interactions, NT x MAT combination recorded the lowest bulk density of 1.139 Mgm^-3^ whilst the highest bulk density of 1.327 Mgm^-3^ was recorded in the DPHR x H plot at the 30–60 cm depth.

**Table 3:** Effect of tillage and weed control methods’ interaction on soil dry bulk density.

	Soil Depth		
Tillage x Weed Control	0–15 cm	15–30 cm	30–60 cm
No Tillage x Hoe	1.162	1.207	1.284
No Tillage x Machete	1.139*	1.207	1.248*
No Tillage x No Weeding	1.165	1.228	1.277
Minimum Tillage x Hoe	1.179	1.23	1.306
Minimum Tillage x Machete	1.170*	1.246	1.285*
Minimum Tillage x No Weeding	1.196	1.267	1.313
Plough + Harrow + Ridging x Hoe	1.179	1.23	1.306
Plough + Harrow + Ridging x Machete	1.156*	1.231	1.270*
Plough + Harrow + Ridging x No Weeding	1.182	1.252	1.299
Deep Tillage + Plough + Harrow + Ridging x Hoe	1.252	1.245	1.327
Deep Tillage +Plough +Harrow +Ridging x Machete	1.229*	1.245	1.291*
Deep Tillage +Plough +Harrow +Ridging x No Weeding	1.255	1.266	1.319

Interpretation: * = Significant Interaction at p < 0.05

Comparatively, for the three soil layers, the lower recorded bulk densities of CsT systems with the weed control interactions NT x (MAT < H < NW) in that order than the CT systems were ascribed to the relatively sufficient amount of vegetal or mulch cover compounded with bioporosity conservation and the minimal soil disturbance of the mechanized weed control methods. That, according to [49], could result in a loose and porous soil structure that could retain and store more moisture. A lower bulk density results in increased porosity, which makes it easier for plants to establish [49]. The lengthy fallow period at the study site and a relatively higher rainfall distribution (Table 2) from September to October 2018 before sampling (which increased moisture retention) is likely a contributing factor to the lower bulk density on the untilled plots [22].

These outcomes are in line with the research findings of [50–52] but contradict those studies described by [42, 53–55], where bulk density is higher in CT practices than NT and RT practices. Jabro et al. [53], in their findings, reported that lower bulk density values under CT and deep tillage were possibly associated with more soil disturbance and loosening which disrupted aggregate stability and continuity of pore geometry, leading to increased soil porosity in CT and Deep tillage than in both NT and RT practices. Changes in seasonal bulk density, as found in the literature, are largely associated with biological and meteorological factors [56] and the temporal variation of soil sampling after tillage [57–59]. Higher bulk density in the CT systems could be explained by the less concentration of organic matter in the PHR x (NW > H > MAT) and DPHR x (NW > H > MAT) plots which lessened stability of soil aggregate, increased compatibility and reduction of pore size distribution. The increase in bulk density was largely experienced in DPHR x (NW > H > MAT) than PHR x (NW > H > MAT), indicating that the soil’s vulnerability to compaction increases as tillage becomes more intense with moderate soil disturbance of the soil surface by the mechanical weed control methods. The higher bulk density in the 30–60 cm lower layer relative to the 0–15 cm upper layer in the DPHR and weed control methods combination were attributed to the settlement and rearrangement of soil particles, consolidation and densification of the soil over time by the use of the subsoiler in breaking the top soil and disaggregating the soil particles.

### 3.2 Effect of tillage and weed control methods’ interaction on moisture content

Table 4 displays the interaction effect of weed control techniques and tillage on soil moisture content. The interaction effects were statistically significant (p < 0.05) in CsT and CT systems with MAT and NW combinations, while CsT and CT with H weeding combinations showed no significant interaction effect (p > 0.05). Again, CsT and CT with MAT weeding treatments recorded the highest moisture content across the studied soil profile, especially for NT x MAT (11.189%) at the 15–30 cm layer; the lowest were observed in the CsT and CT with H weeding interactions, especially for the DPHR x H (8.086%). Comparing the significant interaction effect of the tillage treatments with MAT, the soil moisture content of RT x MAT, PHR x MAT, DPHR x MAT were lower relative to NT x MAT at 11.46%, 12.30% and 17.03% for the 0–15 cm layer and 11.28%, 7.79% and 7.17% for the 15–30 cm layer, respectively. The 15–30 cm middle layer mostly had the highest soil moisture content for both CsT and CT systems. In the plots under CsT methods with all weed control combinations, moisture content was higher in the 0–15 cm upper layer than the bottom (30–60 cm) layer. However, the upper layer had less soil moisture than the bottom layer in plots under CT systems, irrespective of the mechanical weed control method.

**Table 4:** Effect of tillage and weed control methods’ interaction on moisture content.

	Soil Depth		
Tillage x Weed Control	0–15 cm	15–30 cm	30–60 cm
No Tillage x Hoe	9.849	10.746	8.677
No Tillage x Machete	10.361*	11.189*	9.219*
No Tillage x No Weeding	9.883*	10.870*	8.942*
Minimum Tillage x Hoe	8.659	9.484	7.977
Minimum Tillage x Machete	9.171*	9.927*	8.519*
Minimum Tillage x No Weeding	8.694*	9.608*	8.242*
Plough +Harrow +Ridging x Hoe	8.575	9.874	8.712
Plough +Harrow +Ridging x Machete	9.087*	10.317*	9.254*
Plough +Harrow +Ridging x No Weeding	8.609*	9.998*	8.977*
Deep Tillage +Plough +Harrow +Ridging x Hoe	8.086	9.946	8.404
Deep Tillage +Plough +Harrow +Ridging x Machete	8.597*	10.389*	8.945*
Deep Tillage +Plough +Harrow +Ridging x No Weeding	8.120*	10.070*	8.669*

Interpretation: * = Significant Interaction at p < 0.05

The higher moisture content in the soil profile under the CsT and the weed interactions, especially under the MAT control method, were attributed to the retention of biomass residue, minimal mechanical disturbance of vegetal cover, and opening of interconnected pores at the soil surface and soil sheltering against impacting raindrops which led to reduced evaporation losses, increased water infiltration and enhanced available water capacity.

While intense ploughing under CT and weed control interactions pulverise and disrupt the soil surface, increase pore volume and permit water entry (as noted by Appah [60]), retention of moisture can be counterbalanced by the little or no residue retention and deep soil voids which are flow-active, leading to moisture loss by evaporation at the upper layers (15–30 cm) [61] and by percolation at deeper layers [62]. Our study corroborates the results reported by Abdollahi et al. [63] who found higher soil moisture content in the middle layer (12–25 cm) of sandy loam soil on CsT and CT plots and ascribed the higher moisture content to better pore connectivity and higher pore volume, respectively, at that depth. Higher moisture content in the DPHR x (MAT > NW > H) plot by the subsoiler operation appeared to have improved water storage in the lower layer in comparison to lower water content at the upper layer of DPHR and weed control permutations (Table 4), which demonstrated a stronger ability to lessen the impact of water scarcity and waterlogging on crop growth [64]. According to Mudjeci et al. [65] and Hobbs et al. [66], a combination of soil evaporation, effective rainfall, and crop consumptive use or transpiration are major contributing factors to the moisture content changes in the soil profile. Crop yields are most severely impacted by soil moisture and, thus, conservation tillage methods that retain more moisture and increase infiltration rate are important for increasing crop productivity and reducing the disastrous effects of drought [63, 67, 68].

### 3.3 Interaction effect of different tillage systems and weed control methods on soil infiltration rate

**Fig 2:**
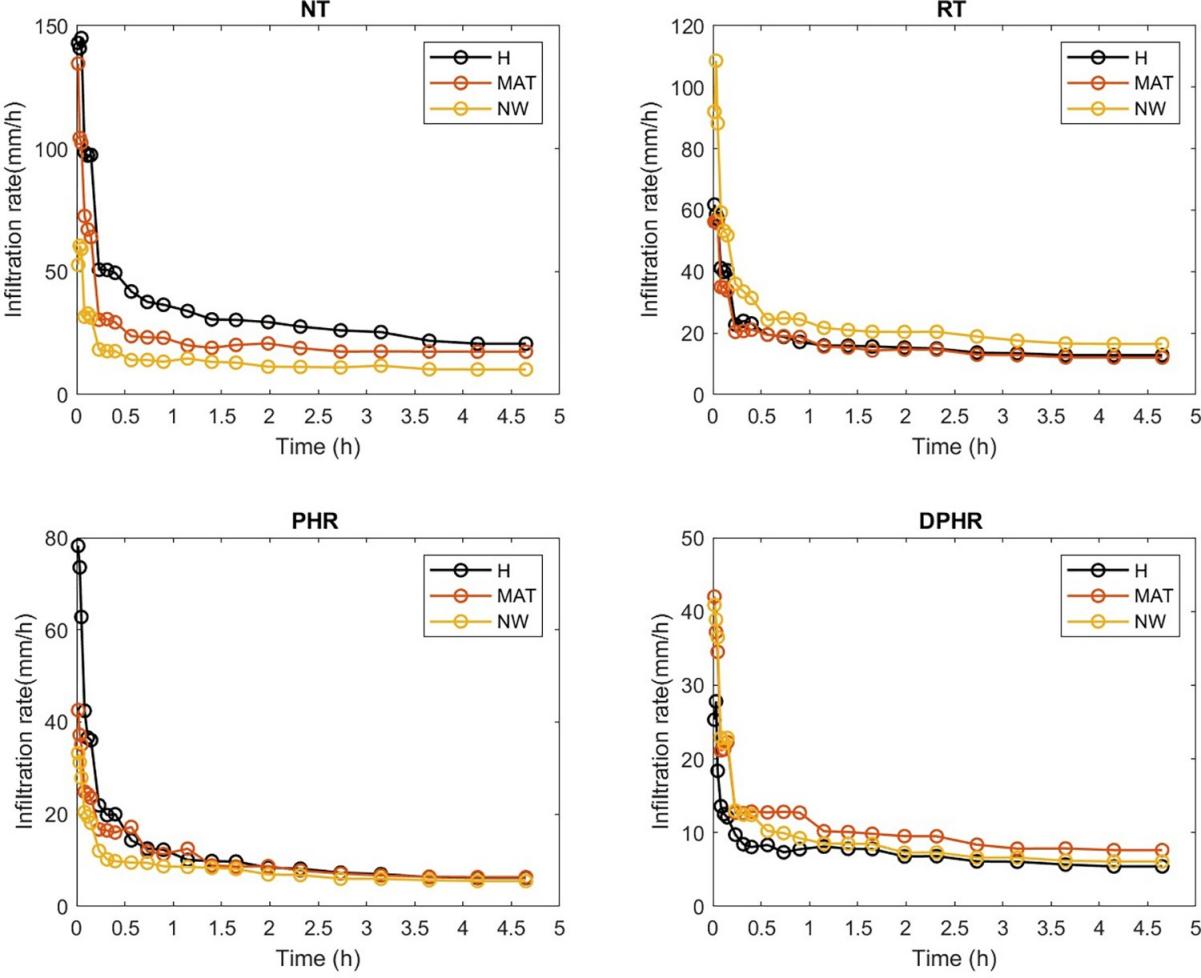
Graph of infiltration capacity showing the interaction between tillage and weed control methods.

At the beginning of the experiment, the measured infiltration rates under the tillage and mechanized weed control methods interactions were high and decayed to a steady infiltration rate (Fig 2). Additionally, there were oscillations in the infiltration rates (Fig 2), particularly during the initial 0.25 hours. This can be ascribed to the existence of soil macropores and inter-pedal voids, the sudden release of trapped air [19, 69], soil disturbance and insufficient pre-wetting [70].

The steady infiltration rate shown in Fig 2 increased in the following order: NT x H (20.7 mm/h) > NT x MAT (17.5 mm/h) > RT x NW (16.4 mm/h) > RT x H (12.8 mm/h) > RT x MAT (12.1 mm/h) > NT x NW (10.2 mm/h) > DPHR x MAT (7.6 mm/h) > PHR x MAT (6.4 mm/h) > PHR x H (6.2 mm/h) > DPHR x NW (6.1 mm/h) > PHR x NW (5.5 mm/h) > DPHR x H (5.4 mm/h).

### 3.4 Estimating parameters for infiltration models

**Fig 3:**
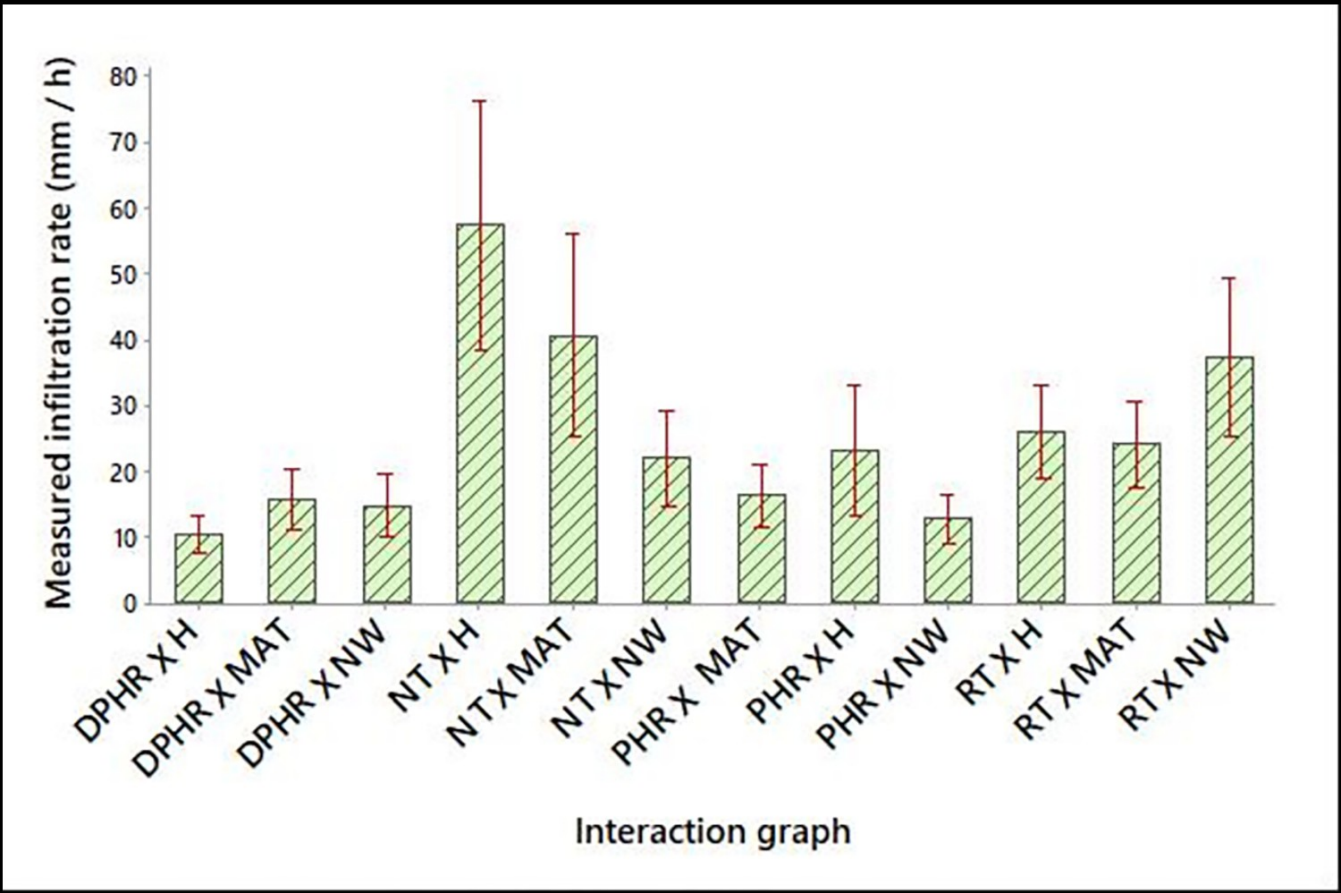
Interaction graph of the combined effect of tillage systems and weed control methods.

Table 5 shows the results of 2 by 3 factorial design to test the combined effect of the different tillage practices and the weed control methods on soil infiltration rates. The results showed that, though the different weed control methods did not significantly (p>0.05) influence soil infiltration rate, when combined with tillage systems in a factorial design, there was a highly significant interaction effect, p < 0.000, *F* (6) = 5.530 (Table 6). From Table 6 and Fig 3, the highest mean infiltration rate was recorded from the NT X H treatment combination followed by NT X MAT and RT X NW combination, whilst the lowest infiltration rate was recorded in the DPHR X H followed by PHR X NW treatment combinations (Table 6, Fig 3). The higher infiltration rates under the CsT and weed interaction treatments could be ascribed to the contribution of vertically oriented flow-active macro-pores and inter-pedal voids made by the increasing activities of surface-feeding macro-organisms and the undisturbed root channels [70, 71] as well as the minimal marked surface disturbance by hoeing [60] and machete weeding at the soil surface. The current findings are in line with earlier studies [22, 72–75] but contradict other researchers [21, 42, 76] who observed higher infiltration rate under CT than CsT tillage. Wang et al. [49] also stated that, in Shanxi Province, CsT practices might increase the steady infiltration rate by 60.9% and delay flow by 12–16 minutes in high rainfall events when compared to CT methods. Significantly higher infiltration rates for two seasons in Zimbabwe (49% and 45% higher) under CsT plots in sandy soils relative to CT systems have been reported by Thierfelder and Wall [77]. Das et al. [78] reported a 26% higher infiltration rate under CsT relative to CT systems in loam to sandy clay loam soil.

**Table 5:** Effect of tillage and weed control methods’ interaction on infiltration rate.

Sum of Squares	Df	Mean Square	F	Sig.
28303.777	3	9434.592	20.692	<0.001
2602.926	2	1301.463	2.854	0.059
15128.992	6	2521.499	5.53	<0.001
114899.574	252	455.951		
160935.269	263			

**Table 6:** Combined effect of tillage systems and weed control methods.

Interaction	Measured Infiltration Rate
NT X H	57.33±9.14 a
NT X MAT	40.56±7.37 b
NT X NW	21.85±3.44 def
RT X H	25.80±3.46 cd
RT X MAT	24.01±3.17 de
RT X NW	37.18±5.80 bc
PHR X H	23.18±4.77 de
PHR X MAT	16.23±2.30 def
PHR X NW	12.62±1.83 ef
DPHR X H	10.24±1.31 f
DPHR X MAT	15.61±2.15 def
DPHR X NW	14.69±2.37 def
*Df*	11
*F-ratio*	9.18
*P-value*	0

Note: *Means that share the same letters along a column are not significantly different at α = 5% (0*.*05) significance level by Fisher’s LSD multiple comparison tests*.

Conservation tillage techniques increase soil aeration, water-holding capacity, infiltration rate which promotes nutrient uptake, root penetration, and strong crop growth by lowering the soil’s resistance to plant roots and agricultural implements penetrating it [79, 80]. The lower infiltration rates under the CT and weed combinations could be ascribed to the mechanical disturbances in the soil aggregate coupled with the burying or removal of above-ground biomass, which exposed the soil to the direct impact of raindrops [81, 82], causing soil particle dispersion; these, over time, lead to a slaked soil structure, blocked pore continuity [22, 83] and consequent surface sealing and crusting. According to Strudley [84], these conflicting results may–in certain circumstances–be explained by the temporal variability of the soil water infiltration rate, which increases quickly after tillage operations but rapidly declines some weeks later, being greater under NT than in CT soils occasionally–even under the initial alternating wetting and drying cycle. Another study by Busari and Salanko [69] also found a higher infiltration rate under CT than CsT practices at the end of the first year but found conservation tillage to be higher at the end of the second year. The difference in their findings for the two-year period was attributed to the fast-draining pores (FDP) fissured by the conventional tillage practice which facilitated infiltration temporarily after tillage. The FDP condensed with time as a result of rearranging of soil aggregate [85], resulting in lower infiltration rates under conventional tillage than under conservation tillage.

Table 7 shows the results of the effect of tillage and weed control interaction on the estimated model parameters. The estimated Kostiakov’s Coefficient *a* increased in this order: NT x H (52.270 mm/h) > NT x MAT (35.34 mm/h) > RT x NW (34.029 mm/h) > RT x H (24.080 mm/h) > RT x MAT (22.54 mm/h) > NT x NW (19.80 mm/h) > PHR x H (18.74 mm/h) > DPHR x MAT (14.63 mm/h) > PHR x MAT (14.34 mm/h) > DPHR x NW (13.30 mm/h) > PHR x NW (11.05 mm/h) > DPHR x H (10.88 mm/h). Moderate soil surface disturbance of the vegetal cover by the mechanical weed control techniques sheltered soil macropores, earthworm activities, and decomposing root channels which improved pore geometry and increased infiltration rate on the NT and RT combinations with H, MAT, and NW plots. This triggered a higher *a* value of the Kostiakov coefficient. The *a* parameter in Kostiakov’s model is an index of infiltration capacity at the beginning of infiltration [86]. Our findings corroborate the research work conducted by Mbagwu [87], who linked higher infiltration to higher Kostiakov’s *a*, whilst lower infiltration was related to lower *a* values. These findings are in accordance with Atta-Darkwa et al. [22] but disagree with the studies conducted by Amami et al. [21].

**Table 7:** Parameters and the goodness of fit statistics of the evaluated models under the tillage and weed control techniques.

	Kostiakov		Philip		Horton		
Treatment	b	a	S	K	i_0_	i_c_	k
NT x H	0.676	52.27	43.61	15.2	86.11	20.69	1.161
NT x MAT	0.626	35.34	36.19	5.29	66.34	17.55	2.312
NT x NW	0.68	19.8	16	6.25	31.2	10.2	1.5
**Mean**	**0.661**	**35.796**	**31.938**	**8.911**	**61.214**	**16.152**	**1.656**
RT x H	0.717	24.08	16.45	10.07	40.96	12.82	1.502
RT x MAT	0.726	22.54	15.04	9.573	36.64	12.07	1.347
RT x NM	0.684	34.03	27.4	10.89	54.97	16.44	1.273
**Mean**	**0.709**	**26.884**	**19.63**	**10.176**	**44.19**	**13.774**	**1.374**
PHR x H	0.566	18.74	23.257	0.871	38.503	6.221	1.359
PHR x MAT	0.68	14.34	11.12	5.564	28.59	6.355	1.352
PHR x NM	0.676	11.05	8.881	4.107	18.94	5.507	1.155
**Mean**	**0.641**	**14.71**	**14.42**	**3.514**	**28.68**	**6.028**	**1.289**
DPHR x H	0.678	10.88	6.295	4.111	13.44	5.425	1.028
DPHR x MAT	0.713	14.63	10.15	5.235	22.92	7.639	1.362
DPHR x NW	0.669	13.3	10	3.47	20.3	6.1	1.48
**Mean**	**0.687**	**12.947**	**8.819**	**4.272**	**18.872**	**6.387**	**1.29**

The study also logged Kostiakov’s exponent coefficient *b* average values which increased in the following order: PHR x ((H, MAT, NW)) (0.641) < NT x ((H, MAT, NW) (0.661)) < DPHR x ((H, MAT, NW)) (0.687) < RT x ((H, MAT, NW) (0.709) treatment. These results agree with the theory of infiltration that put the *b* values to be positive and always less than unity [88]. However, according to Jacka et al. [40], the values of empirical coefficients do not have any physical meaning. Conversely, they reflect the influence of the soil’s physical properties and initial soil moisture condition on infiltration [86, 89]. The parameters predicted by Horton’s model show that the average values of *i*_0_ decreased in this order: NT x (H, > MAT, > NW) (61.21 mm/h) > RT x ((NW > H > MAT) (44.19 mm/h) > PHR x (H, > MAT, > NW) (28.67 mm/h) > DPHR x (MAT > NW > H)) (18.87 mm/h) for the tillage and weed treatment combinations (Table 7). The highest estimated initial infiltration rate *i*_0_ was attributed to the rapid filling of the biopores and worm channels in the CsT plots.

The CsT system had a larger decay *k* constant than the CT system, which describes the abrupt decline in the initial portion of the infiltration curve. These findings are in agreement with the studies conducted by Shukla [38] but differ from the investigations reported by Amami et al. [21].

The values of soil sorptivity, S, obtained by the Philip infiltration model in Table 8 depict an increasing trend in the order of NT x H (43.61 $\mathrm{mm}/\sqrt{h}$) > NT x MAT (36.19 $\mathrm{mm}/\sqrt{h}$) > RT x NW (27.40 $\mathrm{mm}/\sqrt{h}$) > PHR x H (23.257 $\mathrm{mm}/\sqrt{h}$) > RT x H (16.45 $\mathrm{mm}/\sqrt{h}$) NT x NW (16.0 $\mathrm{mm}/\sqrt{h}$) > RT x MAT (15.040 $\mathrm{mm}/\sqrt{h}$) > PHR x MAT (11.12 $\mathrm{mm}/\sqrt{h}$) > DPHR x MAT (10.15 $\mathrm{mm}/\sqrt{h}$) > DPHR x NW (10.0 $\mathrm{mm}/\sqrt{h}$) > PHR x NW (8.881 $\mathrm{mm}/\sqrt{h}$) > DPHR x H (6.295 $\mathrm{mm}/\sqrt{h}$). The average values of K also increased in the order PHR x (H < NW < MAT) (3.514 mm/hr) < DPHR x (NW < H < MAT) (4.272 mm/hr) < NT x (MAT < NW < H) (8.911) < RT x ((MAT < H < NW) (10.18 mm/hr) for the tillage and weed treatment combinations. The sorptivity parameter, S, is the capacity of the soil to transport water by capillary forces, and this changes with initial moisture content. The transmissivity or saturated hydraulic conductivity (Ksat) parameter, K, has been described as the greatest water flow, in completely saturated soil, owing to gravity alone [90]. The higher sorptivity, S, in the NT x H may be influenced by the less disruption of the existing continuity of pores preserved by residue retention and improved by pathways created by burrowing of soil macro-organisms, rapidly increasing water infiltration in CsT than in CT systems [91].

**Table 8:** Statistics of the goodness of fit for estimating cumulative infiltration under various tillage and weed control techniques.

	Kostiakov				Philip				Horton			
Treatment	R^2^	RMSE	MAE	NSE	R^2^	RMSE	MAE	NSE	R^2^	RMSE	MAE	NSE
NT x H	0.9954	2.7	2.06	0.9959	0.9392	9.7	2.06	0.9532	0.964	7.45	6.31	0.9727
NT x MAT	0.9959	1.69	1.3	0.9958	0.9441	6.24	5.75	0.9502	0.991	2.59	2.42	0.9918
NT x NW	0.9987	0.6	0.5	0.9986	0.9645	3.03	2.76	0.9692	0.987	1.83	1.65	0.9891
**Mean**	**0.9967**	**1.66**	**1.29**	**0.9968**	**0.9493**	**6.32**	**3.52**	**0.9575**	**0.981**	**3.96**	**3.46**	**0.9845**
RT x H	0.9993	0.56	0.49	0.9993	0.9609	3.98	3.27	0.9684	0.954	4.33	3.49	0.9646
RT x MAT	0.9998	0.32	0.28	0.9998	0.9724	3.2	2.72	0.9766	0.977	2.9	2.44	0.9813
RT x NM	0.9991	0.85	0.74	0.9991	0.9559	5.68	4.96	0.9637	0.965	5.06	4.23	0.9731
**Mean**	**0.9994**	**0.58**	**0.5**	**0.9994**	**0.9631**	**4.29**	**3.65**	**0.9696**	**0.965**	**4.1**	**3.39**	**0.973**
PHR x H	0.9969	0.71	0.62	0.9971	0.8947	4.11	3.47	0.923	0.936	3.21	2.73	0.9554
PHR x MAT	0.9954	0.8	0.55	0.9959	0.9636	2.25	1.94	0.9695	0.971	2.02	1.67	0.9761
PHR x NM	0.9987	0.34	0.28	0.9987	0.9645	1.79	1.61	0.9688	0.977	1.45	1.25	0.9805
**Mean**	**0.997**	**0.62**	**0.49**	**0.9972**	**0.9409**	**2.72**	**2.34**	**0.9538**	**0.961**	**2.23**	**1.88**	**0.9707**
DPHR x H	0.9947	0.64	0.51	0.9945	0.9978	0.42	0.34	0.9978	0.994	0.68	0.64	0.9948
DPHR x MAT	0.9996	0.28	0.23	0.9996	0.9943	0.94	0.91	0.9944	0.996	0.83	0.78	0.9959
DPHRx NW	0.9994	0.27	0.24	0.9994	0.9985	0.41	0.39	0.9985	0.996	0.68	0.59	0.9961
**Mean**	**0.9979**	**0.4**	**0.33**	**0.9978**	**0.9969**	**0.59**	**0.55**	**0.9969**	**0.995**	**0.73**	**0.67**	**0.9956**

The higher sorptivity values might have resulted from a higher moisture content brought on by the soil’s retention of moisture under the NT system, which decreased evaporation losses and boosted the infiltration rate [22].

The pore continuity in the CsT systems was presumably preserved due to higher pore, greater geometry and aggregate stability; however, the decrease of Ksat by CT operation in the surface soil layer was likely caused by the breakdown of soil aggregates and reduction of soil pores. The higher K in CsT and the weed control interactions increase the transmission properties of the soil [92].

### 3.5 Effectiveness of infiltration models for cumulative infiltration amount estimation

The performance of the three infiltration models was assessed using four statistical indices, namely, Coefficient of Determination (R^2^), Root Mean Squared Error (RMSE), Nash-Sutcliffe Efficiency (NSE) and Mean Absolute Error (MAE)–as well as pictorial indications. The three models’ performance was also assessed when put through various tillage operations and weed control methods. Table 8 displays the RMSE, MAE, NSE, and R^2^ values for the three infiltration models under the twelve tillage and weed control treatments combinations.

Table 8 shows that all infiltration models in sandy clay loam ochrosol soils predicted cumulative infiltration amount with extremely high fitting ability under various tillage operations and weed control methods combinations, although the Kostiakov model stood out, followed by the Horton and the Philip models, in that order. The goodness of fit statistical fitting ability of the Kostiakov model exhibited extremely high accuracy with the highest R^2^ and NSE mean values (R^2^: 0.9967–0.9994 and NSE: 0.9968–0.9994) for treatments RT x MAT > DPHR x MAT > DPHR x NW interactions, and lowest errors (RMSE: 0.40–1.66 mm and MAE: 0.33–1.29 mm) for treatments DPHR x MAT > DPHR x NW > RT x MAT interactions, respectively. The Horton model also performed reasonably well with higher mean values of R^2^ and NSE (R^2^: 0.9611–0.9951 and NSE: 0.9707–0.9956) for treatments DPHR x NW > DPHR x MAT > RT x MAT interactions and lower errors (RMSE: 0.73–4.10 mm and MAE: 0.67–3.46 mm) for treatments DPHR x NW > DPHR x H > DPHR x MAT interactions. Philip’s model was also capable of predicting the cumulative infiltration with high R^2^ and NSE mean values (R^2^: 0.9409–0.9969 and NSE: 0.9538–0.9969) for treatments DPHR x NW > DPHR x H > DPHR x MAT interaction and low errors (RMSE: 0.59–6.32 mm and MAE: 0.55–3.65 mm) for DPHR x H > DPHR x NW > DPHR x MAT interaction. In evaluating the performance of the selected models, the present study cohered with the observations of de Almeida et al. [18]. The results show that the Philip model performed satisfactorily with the largest RMSE and MAE and the lowest NSE and R^2^, lagging behind the Kostiakov and the Horton models. With the combined assessment of RMSE, MAE, R^2^ and NSE goodness of fit, the Kostiakov model was found to be in close agreement between the observed and predicted cumulative infiltration amount in the studied sandy clay loam soil for DPHR x MAT > DPHR x NW > DPHR x H > RT x MAT > PHR x NW > RT x H > PHR x MAT > PHR x H > RT x NW > NT x NW > NT x MAT > NT x H interactions. Contrary to these findings, Amami et al. [21] reported that the Philip model closely followed the measured cumulative infiltration amount in the studied sandy clay loam soil. According to Shukla [38], the use of empirical models (Kostiakov and Horton) is verified under experimental conditions and provides better results than the theoretical-based Philip model. This assertion confirms the outcomes of the present study in which the empirical model outperformed the theoretical-based model. The least error (MAE and RMSE) values assessed under the CT and weed control operations prove the dependability of these models for accurately estimating the cumulative infiltration amount of the soil (Table 8). The comparatively high MAE and RMSE values obtained under the CsT and weed management measures, however, show that vegetation cover has a negative impact on the simulation of soil water infiltration [41]. These results affirm the impact of residue retention of previous crops and plant cover on soil water infiltration. This presupposes that vegetation cover affects infiltration by intercepting and storing rainfall, increasing the overall porosity, and offering barriers to the soil surface, thereby restricting surface runoff, providing adequate protection to dissipate the impact of the kinetic energy of raindrops, and altering soil characteristics [9, 82, 93] and, consequently, the soil water infiltration [41, 94]. One of the main causes of the pronounced effect of CT (burying of residue) on the simulation’s accuracy is that the infiltration models were often created for bare soils.

**Fig 4:**
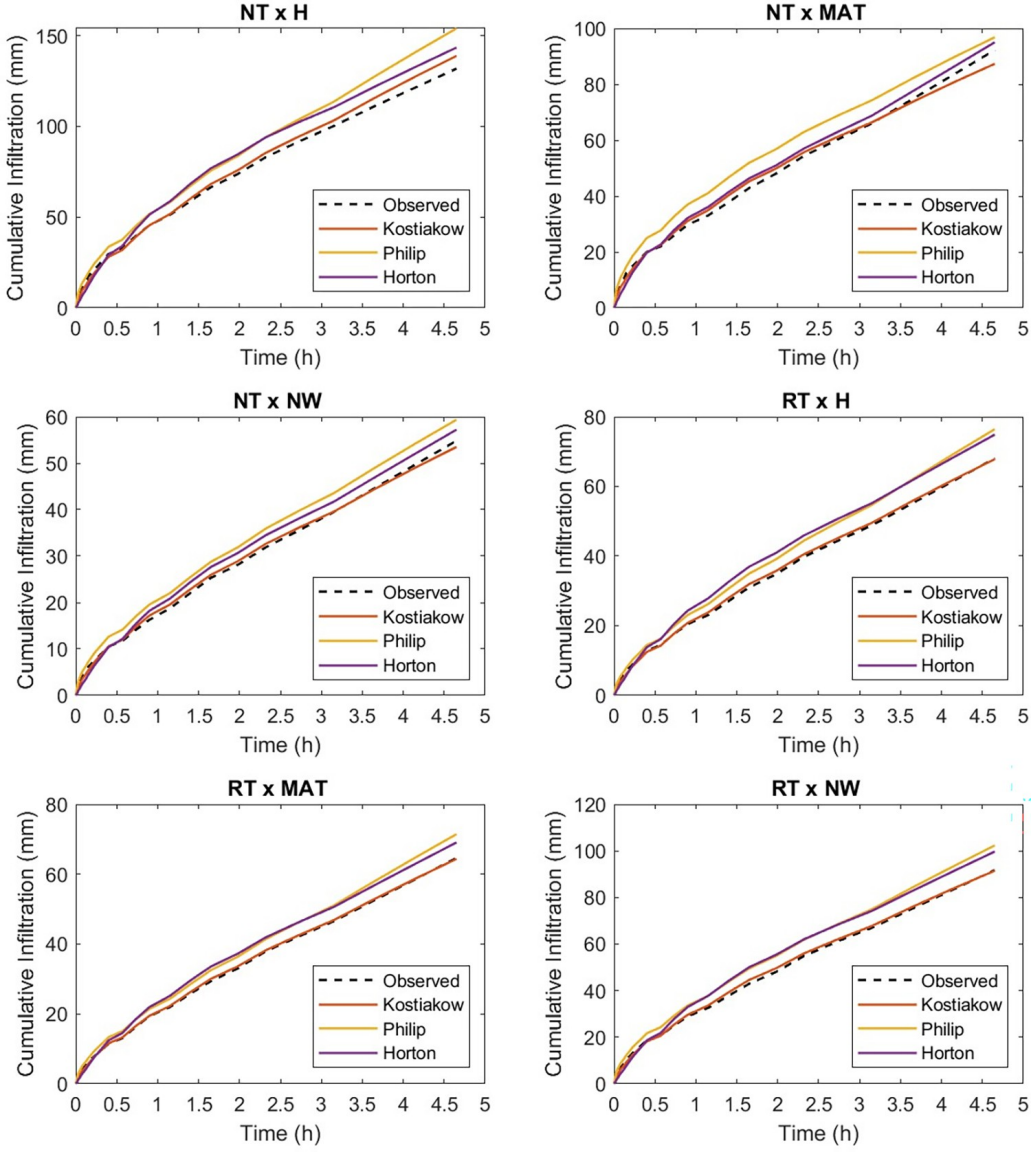
Graphical representation of the predicted and observed cumulative infiltration under the CsT systems and weed control methods’ interactions.

**Fig 5:**
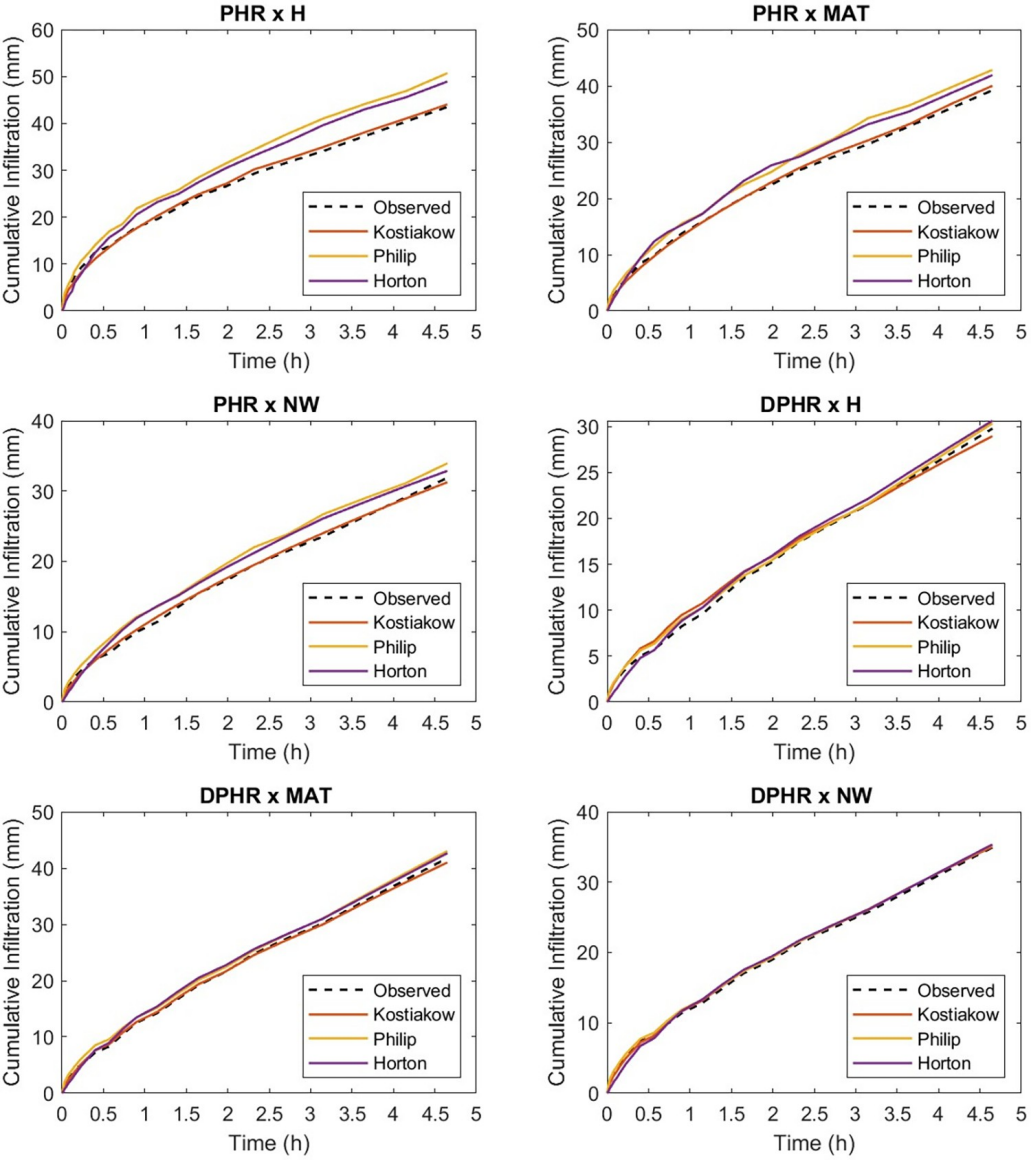
Graphical representation of the predicted and observed cumulative infiltration under the CT systems and weed control methods’ interactions.

Figs 4 and 5 show the graphics of the predictableness of the three infiltrations models for cumulative infiltration amount. Both the Horton and the Philip models closely followed the measured values by the infiltrometer cylinder for the initial 30 min but overpredicted the cumulative infiltration amount till the end of the field test, while the Kostiakov model was found to be in close agreement with the observed cumulative infiltration amount at the beginning and till the end of the experiment under the NT and weed interaction condition. Throughout the experiment, the performance of the Kostiakov model was closely related to the measured data by the infiltrometer cylinder under the RT and weed interaction operations. Nonetheless, both the Horton and the Philip models overestimated and underestimated the infiltration, respectively, after the initial 60 minutes of the infiltration test but overpredicted the infiltration until the end of the field test. For the PHR and weed treatment interaction, the Horton model underpredicted and the Philip model overpredicted the cumulative infiltration amount during the initial 30 minutes, but both overpredicted the infiltration towards the end, while the curve of the Kostiakov model remained the same with the infiltrometer cylinder records from the beginning and till the end of the experiment. Both the Horton and the Philip models underestimated and overestimated the observed values during the initial 60 minutes, but the two models drew level with the curve of the Kostiakov model by closely following the measured cumulative infiltration amount from the first hour till the end of the field test under the DPHR and weed interaction treatments. The examination of Figs 4 and 5 revealed that the Kostiakov model was superior to the Philip and the Horton models because, for all tillage treatments and weed control method interactions, especially DPHR x NW, the curves became almost identical.

The two-factor factorial ANOVA results presented in Table 10 show that the tillage method and weed control mechanism adopted in the study, as well as their interaction, is significant (p < 0.05) to the level of absolute discrepancy observed in all three models.

As observed in Table 9, there were significant effects of tillage and weed control methods in all models. However, the mean differences in the Kostiakov estimates are relatively low compared to estimates of the Philips and the Horton models for both weed control and tillage methods. The Philip model recorded the highest mean absolute difference in the tillage method under No Tillage (NT) whiles DPHR had the least mean difference. With regard to the weed control method, the use of hoe (H) recorded the highest mean difference than those of MAT and No Weeding (NW). In the Horton model, the highest difference was recorded for the No Tillage (NT) mechanism compared to the other weed control mechanisms. And, for the weed control method, the use of hoe (H) recorded the highest absolute difference infiltration values than MAT and NW. It is also worth noting that the use of DPHR tillage method was associated with the minimum observed absolute difference across three prediction models. Concerning weed control techniques, No Weeding (NW) mechanism under the Kostiakov and the Philip models was associated with the minimum observed differences, whilst the use of a machete (MAT) had the lowest difference for the Horton model.

**Table 9:** ANOVA for discrepancies models.

Source	Kosti	Philip	Hort
Tillage	< 0.001*	< 0.001*	< 0.001*
Weed Control	< 0.001*	.001*	< 0.001*
Tillage*Weed Control	< 0.001*	< 0.001*	< 0.001*

Interpretation: *Significant at *α* = 0.05.

**Fig 6:**
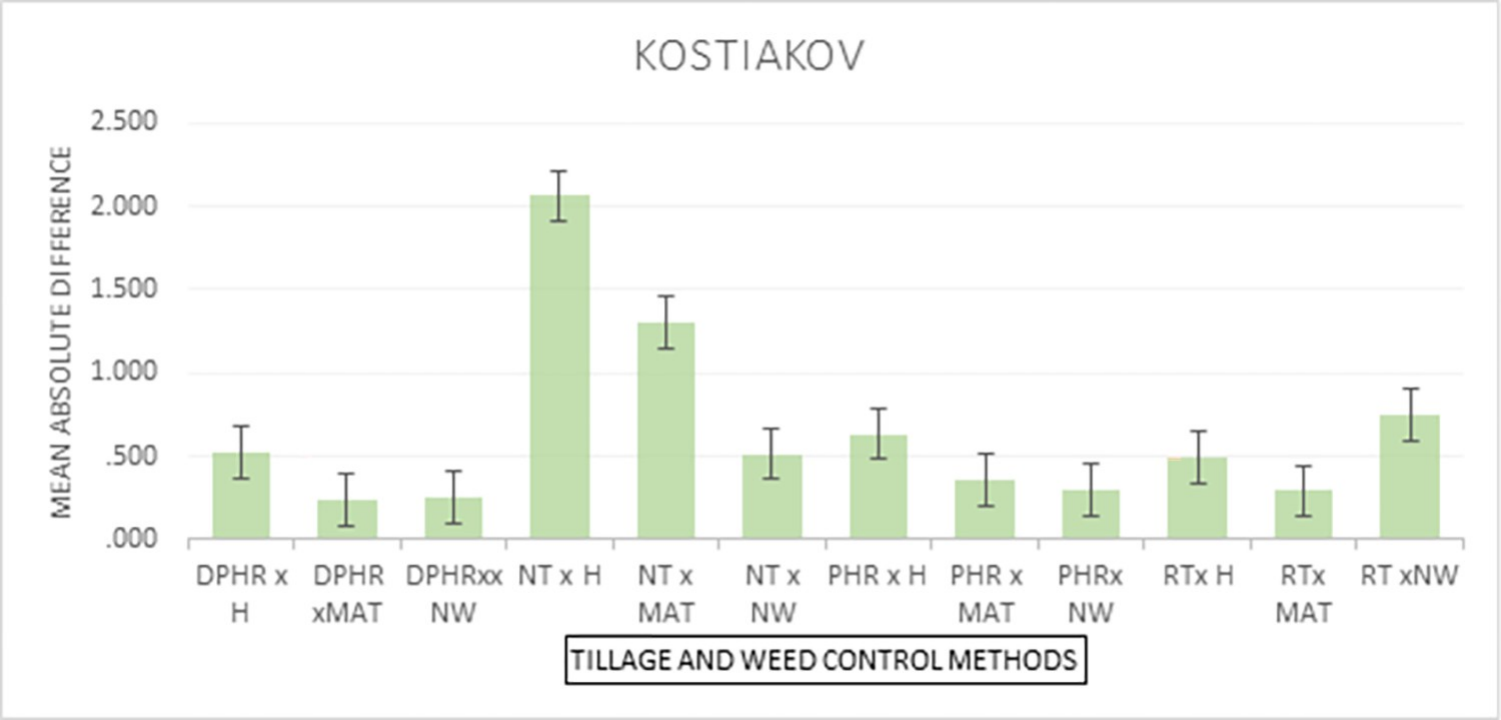
Kostiakov’s model interaction effects of weed control and tillage methods.

**Fig 7:**
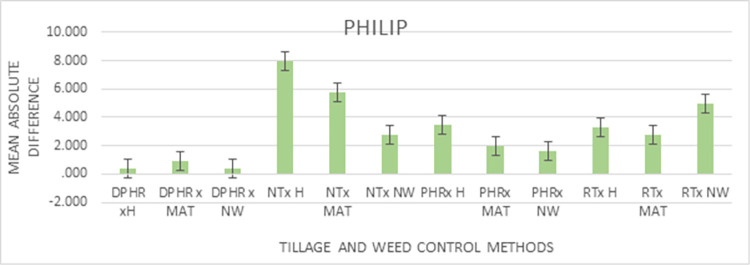
Philip’s Model Interaction effects of weed control and tillage methods.

**Fig 8:**
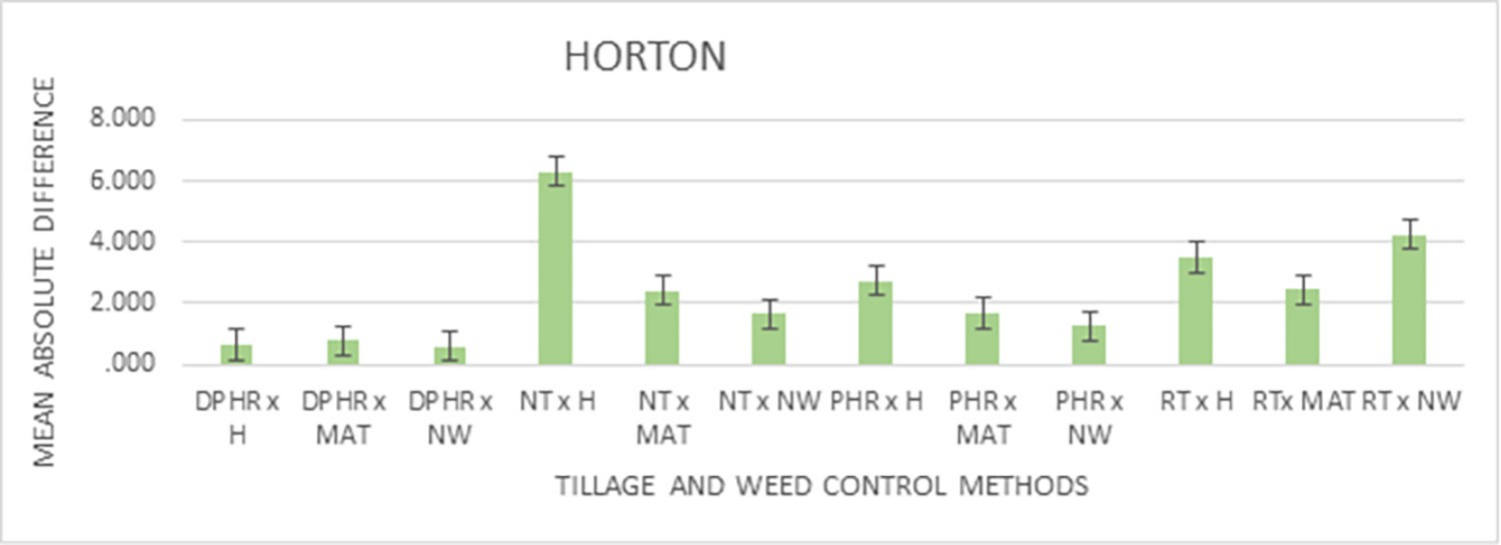
Horton’s Model Interaction effects of weed control and tillage methods.

According to the results presented in Table 10, the interaction effects under all models were significantly associated with the observed differences in the cumulative infiltration values. The interaction effects results in Table 10 show that the lowest absolute differences were recorded in the use of DPHR with a machete with a mean difference of 0.237 mm, and in the use of DPHR with no weeding (NW) with a mean difference of 0.252 mm. The highest difference was observed under the Philips model with the use of hoe (H) for weed control and No Tillage (NT) for tilling the soil, with a mean absolute difference of 7.928 mm. Graphical representations for the mean interaction effects for weed control and tillage methods effects on the observed differences for the various models are shown in Figs 6–8.

**Table 10:** Main and interaction effects of factors on discrepancies.

	Kostiakov	Philip	Horton
**Tillage**			
NT	1.295a	5.487a	3.470a
RT	0.511b	3.659b	3.397a
PHR	0.428b	2.348c	1.897b
DPHR	0.337b	0.559d	0.677c
**Weed Control**	** **	** **	** **
H	0.930a	3.759a	3.302a
MAT	0.547b	2.841b	1.837b
NW	0.452b	2.439b	1.942b
**Tillage and Weed Control**	** **	** **	** **
NT x H	2.065a	7.928a	6.316a
NT x MAT	1.305b	5.763b	2.430b
NT x NW	0.515c	2.769c	1.664c
RT x H	0.497d	3.280c	3.503b
RT x MAT	0.290c	2.729c	2.447b
RT x NW	0.746c	4.967b	4.241b
PHR x H	0.634c	3.476c	2.744b
PHR X MAT	0.355d	1.946d	1.684c
PHR x NW	0.295d	1.621d	1.264c
DPHR x H	0.524c	0.354d	0.646c
DPHR x MAT	0.237d	0.924d	0.788c
DPHR x NW	0.252d	0.399d	0.598c

Interpretation: Different letters imply significantly different mean effects at *α* = 0.05.

Fig 6 shows the interaction of weed control and tillage mechanisms’ effects on the Kostiakov model. The plot (Fig 6) indicates that the use of DPHR with No Weeding (NW) or DPHR with a machete (MAT) are linked with the low differences in infiltration values. The use of hoe (H) or No Weeding (NW) with No Tillage (NT) resulted in high absolute differences.

The interaction effects of weed control and tillage mechanisms for the Philip model are presented in Fig 7. Minimum differences were observed for the use of DPHR tillage method with either No Weeding (NW) or hoe (H). Similar to the results in the Kostiakov model, No Tillage (NT) with the use of a hoe or machete recorded the highest differences, relative to the other tillage and weed control methods.

Fig 8 presents the interaction effects of the Horton model under various tillage and weed control mechanisms. The highest mean absolute difference was observed in the No Tillage with hoe followed by the Reduced Tillage (RT) with No Weeding (NW). However, the low difference(s) was/were associated with DPHR with No Weeding as a means of weed control for agricultural purposes.

The efficiency of the fitting models (Kostiakov *>* Horton *>* Philip) highly prioritised soil cultivation and farm management under these orderly treatments: DPHR x MAT > DPHR x NW > DPHR x H > RT x MAT > PHR x NW > PHR x MAT > NT x NW > RT x MAT > PHR x H > RT H > NT x MAT > RT x NW > NT x H. The trend shows that the increase in tillage intensity and the decrease in weed management intensity induce the quality of the estimation process and vice versa. The study identified the use of a machete with DPHR under the Kostiakov model as the best option for modelling the infiltration characteristics of the sandy clay loam ochrosols.

## 4.0 Conclusion

Infiltration characteristics and the efficacy of the infiltration models (Kostiakov, Horton, and Philip) have been evaluated under the interaction effect of tillage and weed control methods. The results show that NT x MAT tillage and weed control interactions recorded the lowest bulk density whilst the highest bulk density was recorded in the DPHR x H plot at the 30–60 cm depth. Comparing the significant interaction effect of the tillage treatments with MAT, the soil moisture content of RT x MAT, PHR x MAT, DPHR x MAT were lower relative to NT x MAT and decreased by 11.46%, 12.30% and 17.03% for the 0–15 cm layer and 11.28%, 7.79% and 7.17% for the 15–30 cm layer, respectively. Moreover, the highest mean infiltration rate was recorded from the NT X H treatment combination followed by NT X MAT and RT X NW combination, whilst the lowest infiltration rate was recorded in the DPHR X H and PHR X NW treatment combinations. Regarding the combined assessment of RMSE, MAE, R^2^, and NSE goodness of fit, the Kostiakov model was found to be in close agreement with the measured cumulative infiltration amount in the studied sandy clay loam soil for DPHR x MAT > DPHR x NW > DPHR x H > RT x MAT > PHR x NW > RT x H > PHR x MAT > PHR x H > RT x NW > NT x NW > NT x MAT > NT x H interactions. Furthermore, the interaction effects results show that the lowest absolute difference between the measured and estimated cumulative infiltration rate was recorded under the DPHR with a machete (MAT) with a mean difference of 0.237 mm, and DPHR with No Weeding (NW) with a mean difference of 0.252 mm for the Kostiakov model. The highest difference was observed under the Philip model with the use of hoe (H) for weed control and No Tillage (NT) for tilling the soil, with a mean absolute difference of 7.928 mm. Although all the models for estimating cumulative infiltration amount performed well under the different weed control and tillage methods, the Kostiakov model estimates were most similar to the actual measured cumulative infiltration. The study identified the use of a machete (MAT) with DPHR under the Kostiakov model as the best land management option for modelling the cumulative infiltration of the sandy clay loam ochrosols. On the soil infiltration characteristics, the decrease in tillage intensity and the increase in weed management intensity increase the infiltration and vice versa. However, the increase in tillage intensity and the decrease in weed management intensity induce the quality of the estimation process and vice versa.

It can be concluded from our findings that both conventional tillage (CT) and minimal weed control mechanism combinations have a synergistic positive effect on the fitting ability and the accuracy of the estimation models.

## Supporting information

S1 File (ZIP)
